# 4-Nitro­phenyl 1-naphthoate

**DOI:** 10.1107/S1600536810011736

**Published:** 2010-04-02

**Authors:** Uzma Bibi, Humaira M. Siddiqi, Zareen Akhter, Michael Bolte

**Affiliations:** a Department of Chemistry, Quaid-I-Azam University, Islamabad 45320, Pakistan; b Institut für Anorganische Chemie, J.-W.-Goethe-Universität Frankfurt, Max-von-Laue-Strasse 7, 60438 Frankfurt/Main, Germany

## Abstract

In the title compound, C_17_H_11_NO_4_, the dihedral angle between the two benzene rings is 8.66 (3)°. The nitro group is twisted by 4.51 (9)° out of the plane of the aromatic ring to which it is attached. The presence of inter­molecular C—H⋯O contacts in the crystal structure leads to the formation of chains along the *c* axis.

## Related literature

For biological and synthetic background, see: Bezerra-Netto *et al.* (2006 ▶); Bibi *et al.* (2009 ▶); Kumarraja & Pitchumani (2004 ▶); Selvakumar *et al.* (2002 ▶); Tafesh & Weiguny (1996 ▶).
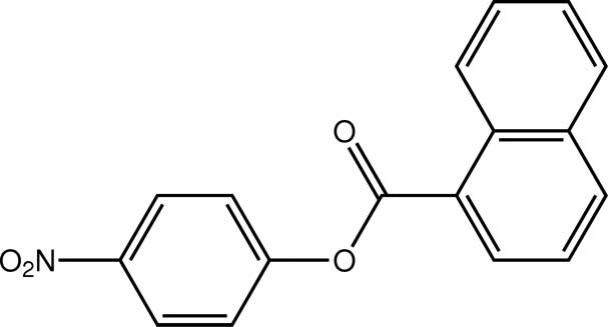


## Experimental

### 

#### Crystal data

C_17_H_11_NO_4_
*M* *_r_* = 293.27
Monoclinic, 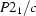

*a* = 7.2049 (6) Å
*b* = 12.8175 (8) Å
*c* = 14.7838 (14) Å
β = 99.006 (7)°
*V* = 1348.44 (19) Å^3^
*Z* = 4
Mo *K*α radiation
μ = 0.10 mm^−1^
*T* = 173 K
0.25 × 0.21 × 0.21 mm

#### Data collection

Stoe IPDS-II two-circle diffractometer
10239 measured reflections
2508 independent reflections
1928 reflections with *I* > 2σ(*I*)
*R*_int_ = 0.040

#### Refinement

*R*[*F* ^2^ > 2σ(*F* ^2^)] = 0.034
*wR*(*F* ^2^) = 0.088
*S* = 0.96
2508 reflections
200 parameters
H-atom parameters constrained
Δρ_max_ = 0.26 e Å^−3^
Δρ_min_ = −0.20 e Å^−3^

### 

Data collection: *X-AREA* (Stoe & Cie, 2001 ▶); cell refinement: *X-AREA*; data reduction: *X-AREA*; program(s) used to solve structure: *SHELXS97* (Sheldrick, 2008 ▶); program(s) used to refine structure: *SHELXL97* (Sheldrick, 2008 ▶); molecular graphics: *XP* (Sheldrick, 2008 ▶); software used to prepare material for publication: *SHELXL97*.

## Supplementary Material

Crystal structure: contains datablocks I, global. DOI: 10.1107/S1600536810011736/tk2650sup1.cif

Structure factors: contains datablocks I. DOI: 10.1107/S1600536810011736/tk2650Isup2.hkl

Additional supplementary materials:  crystallographic information; 3D view; checkCIF report

## Figures and Tables

**Table 1:** Hydrogen-bond geometry (Å, °)

*D*—H⋯*A*	*D*—H	H⋯*A*	*D*⋯*A*	*D*—H⋯*A*
C18—H18⋯O2^i^	0.95	2.45	3.3728 (18)	164

Symmetry code: (i) 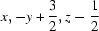.

**Fig. 1.**
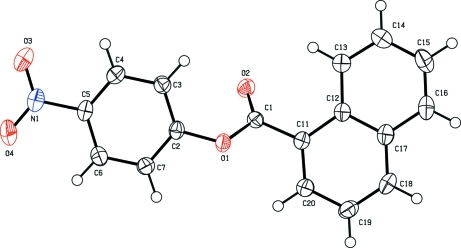
Molecular structure of the title compound. Displacement ellipsoids are drawn at the 50% probability level.

## supplementary crystallographic information

### Comment

The ability of non-steroidal anti–inflammatory drugs (NSAID's) to modulate the pain, inflammation and fever make them attractive drugs (Bezerra-Netto *et al.*, 2006). Aromatic esters containing a nitro-substituted phenyl ring form a medicinally important class of NSAID's. These can be used as starting materials for the preparation of several analgesic and anti–inflammatory drugs, and some of them are potential intermediates in natural product syntheses (Selvakumar *et al.*, 2002). Nitro compounds can be reduced to give amines which are important synthons for preparing a large number of technologically important materials (Kumarraja *et al.*, 2004; Tafesh *et al.*, 1996). In continuation of studies on related compounds (Bibi *et al.*, 2009), the title compound is reported herein.

A perspective view of the title compound is shown in Fig. 1. The dihedral angle formed between the two aromatic ring systems is 8.66 (3)°. The nitro group is twisted by only 4.51 (9)° out of the plane of the aromatic ring to which it is attached. The crystal structure is stabilized by C—H···O contacts, Table 1.

### Experimental

1-Naphthoic acid (1.5 g, 1 mol) was taken in a 100 ml two neck round bottom flask and warmed on a water bath to 323 K. An excess of dry thionyl chloride was added slowly with stirring. Drops (2-3) of DMF were added and the mixture was refluxed for about 50-60 minutes at 343 K. After the completion of the reaction, excess thionyl chloride was removed by repeated evaporations at reduced pressure. 4-Nitrophenol (1.5 g, 0.0065 mol) was dissolved in dry dichloromethane containing triethyl amine at room temperature. The acid chloride was added drop-wise with constant stirring at room temperature for half an hour. The reaction mixture was heated gently for 30 minutes under anhydrous condition and then the solution was poured with constant stirring into cold water (20 ml ). Excess triethyl amine was destroyed by adding the cold dilute HCl solution. The reaction was monitored by TLC using ethyl acetate:n-hexane (1:1). After the completion of reaction the oily product was allowed to settle down and the supernatant liquid was decanted. The product was stirred well with distilled water and extracted with ethyl acetate (3 x 40 ml). Washing was done with 5% NaHCO_3_ solution to remove unreacted acid and the extract was dried over anhydrous Na_2_SO_4_, filtered, and concentrated on a rotary evaporator. The ester soon solidified and was filtered. The title compound was recrystallized from n-hexane (Yield 36.5 %, m.pt. 385–393 K)

### Refinement

H atoms were found in a difference map, but they were refined with fixed individual isotropic displacement parameters [*U*_iso_(H) = 1.2*U*_eq_(C)] using a riding model, with C—H = 0.95 Å.

### Crystal data

**Table:**

C_17_H_11_NO_4_	*F*(000) = 608
*M**_r_* = 293.27	*D*_x_ = 1.445 Mg m^−^^3^
Monoclinic, *P*2_1_/*c*	Mo *K*α radiation, λ = 0.71073 Å
Hall symbol: -P 2ybc	Cell parameters from 8709 reflections
*a* = 7.2049 (6) Å	θ = 3.3–26.0°
*b* = 12.8175 (8) Å	µ = 0.10 mm^−^^1^
*c* = 14.7838 (14) Å	*T* = 173 K
β = 99.006 (7)°	Block, colourless
*V* = 1348.44 (19) Å^3^	0.25 × 0.21 × 0.21 mm
*Z* = 4	

### Data collection

**Table:**

Stoe IPDS-II two-circle diffractometer	1928 reflections with *I* > 2σ(*I*)
Radiation source: fine-focus sealed tube	*R*_int_ = 0.040
graphite	θ_max_ = 25.6°, θ_min_ = 3.3°
ω scans	*h* = −8→8
10239 measured reflections	*k* = −15→15
2508 independent reflections	*l* = −13→17

### Refinement

**Table:**

Refinement on *F*^2^	Secondary atom site location: difference Fourier map
Least-squares matrix: full	Hydrogen site location: inferred from neighbouring sites
*R*[*F*^2^ > 2σ(*F*^2^)] = 0.034	H-atom parameters constrained
*wR*(*F*^2^) = 0.088	*w* = 1/[σ^2^(*F*_o_^2^) + (0.0582*P*)^2^] where *P* = (*F*_o_^2^ + 2*F*_c_^2^)/3
*S* = 0.96	(Δ/σ)_max_ < 0.001
2508 reflections	Δρ_max_ = 0.26 e Å^−^^3^
200 parameters	Δρ_min_ = −0.20 e Å^−^^3^
0 restraints	Extinction correction: *SHELXL97* (Sheldrick, 2008), Fc^*^=kFc[1+0.001xFc^2^λ^3^/sin(2θ)]^-1/4^
Primary atom site location: structure-invariant direct methods	Extinction coefficient: 0.0103 (16)

### Special details

**Table:**

Geometry. All esds (except the esd in the dihedral angle between two l.s. planes) are estimated using the full covariance matrix. The cell esds are taken into account individually in the estimation of esds in distances, angles and torsion angles; correlations between esds in cell parameters are only used when they are defined by crystal symmetry. An approximate (isotropic) treatment of cell esds is used for estimating esds involving l.s. planes.
Refinement. Refinement of *F*^2^ against ALL reflections. The weighted *R*-factor wR and goodness of fit *S* are based on *F*^2^, conventional *R*-factors *R* are based on *F*, with *F* set to zero for negative *F*^2^. The threshold expression of *F*^2^ > σ(*F*^2^) is used only for calculating *R*-factors(gt) etc. and is not relevant to the choice of reflections for refinement. *R*-factors based on *F*^2^ are statistically about twice as large as those based on *F*, and *R*- factors based on ALL data will be even larger.

### Fractional atomic coordinates and isotropic or equivalent isotropic displacement parameters (Å^2^)

**Table:**

	*x*	*y*	*z*	*U*_iso_*/*U*_eq_	
N1	0.36959 (17)	0.15975 (9)	0.73346 (9)	0.0357 (3)	
O1	0.28241 (13)	0.47946 (6)	0.47417 (6)	0.0264 (2)	
O2	0.14518 (13)	0.59160 (7)	0.56191 (7)	0.0303 (2)	
O3	0.42873 (18)	0.18063 (9)	0.81407 (8)	0.0526 (3)	
O4	0.33027 (18)	0.07066 (8)	0.70625 (9)	0.0519 (3)	
C1	0.19783 (17)	0.57191 (9)	0.49057 (9)	0.0239 (3)	
C2	0.30357 (17)	0.40273 (9)	0.54233 (9)	0.0239 (3)	
C3	0.38432 (18)	0.42424 (10)	0.63163 (10)	0.0275 (3)	
H3	0.4249	0.4929	0.6491	0.033*	
C4	0.40489 (18)	0.34404 (10)	0.69505 (10)	0.0291 (3)	
H4	0.4579	0.3568	0.7570	0.035*	
C5	0.34691 (18)	0.24492 (10)	0.66653 (9)	0.0272 (3)	
C6	0.27048 (19)	0.22251 (10)	0.57696 (10)	0.0301 (3)	
H6	0.2335	0.1534	0.5592	0.036*	
C7	0.24893 (19)	0.30293 (10)	0.51368 (10)	0.0280 (3)	
H7	0.1975	0.2899	0.4516	0.034*	
C11	0.17730 (17)	0.63938 (10)	0.40772 (9)	0.0239 (3)	
C12	0.20200 (17)	0.75036 (9)	0.41572 (9)	0.0250 (3)	
C13	0.2570 (2)	0.80306 (10)	0.49996 (10)	0.0323 (3)	
H13	0.2770	0.7646	0.5556	0.039*	
C14	0.2816 (2)	0.90913 (11)	0.50176 (12)	0.0426 (4)	
H14	0.3169	0.9436	0.5588	0.051*	
C15	0.2553 (2)	0.96756 (11)	0.42033 (13)	0.0460 (4)	
H15	0.2738	1.0410	0.4226	0.055*	
C16	0.2035 (2)	0.91929 (11)	0.33809 (12)	0.0380 (4)	
H16	0.1867	0.9594	0.2834	0.046*	
C17	0.17434 (18)	0.80981 (10)	0.33332 (10)	0.0286 (3)	
C18	0.12013 (19)	0.75958 (11)	0.24776 (10)	0.0322 (3)	
H18	0.0998	0.8000	0.1932	0.039*	
C19	0.0969 (2)	0.65407 (11)	0.24282 (10)	0.0334 (3)	
H19	0.0601	0.6213	0.1851	0.040*	
C20	0.12749 (19)	0.59370 (10)	0.32339 (10)	0.0288 (3)	
H20	0.1134	0.5201	0.3193	0.035*	

### Atomic displacement parameters (Å^2^)

**Table:**

	*U*^11^	*U*^22^	*U*^33^	*U*^12^	*U*^13^	*U*^23^
N1	0.0365 (7)	0.0352 (7)	0.0361 (8)	0.0029 (5)	0.0078 (5)	0.0115 (5)
O1	0.0335 (5)	0.0227 (4)	0.0240 (5)	0.0043 (4)	0.0081 (4)	0.0044 (4)
O2	0.0387 (6)	0.0295 (5)	0.0240 (5)	0.0049 (4)	0.0089 (4)	0.0020 (4)
O3	0.0747 (9)	0.0495 (7)	0.0318 (7)	0.0025 (6)	0.0026 (6)	0.0140 (5)
O4	0.0705 (8)	0.0296 (6)	0.0539 (8)	−0.0058 (5)	0.0045 (6)	0.0137 (5)
C1	0.0237 (6)	0.0228 (6)	0.0251 (7)	−0.0011 (5)	0.0036 (5)	−0.0006 (5)
C2	0.0236 (6)	0.0241 (6)	0.0252 (7)	0.0031 (5)	0.0075 (5)	0.0043 (5)
C3	0.0278 (7)	0.0253 (6)	0.0289 (8)	−0.0011 (5)	0.0033 (5)	0.0004 (5)
C4	0.0277 (7)	0.0333 (7)	0.0256 (8)	0.0022 (5)	0.0018 (6)	0.0022 (6)
C5	0.0253 (7)	0.0280 (7)	0.0291 (8)	0.0039 (5)	0.0070 (5)	0.0090 (5)
C6	0.0316 (7)	0.0233 (6)	0.0350 (9)	0.0002 (5)	0.0045 (6)	0.0016 (6)
C7	0.0303 (7)	0.0275 (7)	0.0256 (7)	0.0026 (5)	0.0026 (5)	−0.0007 (5)
C11	0.0219 (6)	0.0259 (6)	0.0246 (7)	0.0012 (5)	0.0053 (5)	0.0029 (5)
C12	0.0218 (6)	0.0254 (6)	0.0282 (8)	0.0015 (5)	0.0051 (5)	0.0029 (5)
C13	0.0368 (8)	0.0296 (7)	0.0299 (8)	−0.0010 (5)	0.0033 (6)	0.0004 (6)
C14	0.0546 (10)	0.0309 (8)	0.0409 (10)	−0.0040 (6)	0.0033 (7)	−0.0061 (6)
C15	0.0567 (11)	0.0247 (7)	0.0561 (12)	−0.0042 (7)	0.0070 (8)	0.0015 (7)
C16	0.0397 (8)	0.0300 (7)	0.0441 (10)	0.0021 (6)	0.0061 (7)	0.0131 (6)
C17	0.0235 (6)	0.0293 (7)	0.0333 (8)	0.0023 (5)	0.0058 (6)	0.0063 (6)
C18	0.0315 (7)	0.0386 (8)	0.0265 (8)	0.0016 (6)	0.0042 (6)	0.0111 (6)
C19	0.0372 (8)	0.0396 (8)	0.0228 (7)	−0.0025 (6)	0.0026 (6)	0.0010 (6)
C20	0.0314 (7)	0.0281 (6)	0.0271 (8)	−0.0013 (5)	0.0050 (6)	0.0009 (5)

### Geometric parameters (Å, °)

**Table:**

N1—O4	1.2290 (16)	C11—C12	1.4362 (18)
N1—O3	1.2307 (18)	C12—C13	1.418 (2)
N1—C5	1.4651 (17)	C12—C17	1.4242 (19)
O1—C1	1.3713 (15)	C13—C14	1.371 (2)
O1—C2	1.3993 (15)	C13—H13	0.9500
O2—C1	1.2022 (17)	C14—C15	1.405 (2)
C1—C11	1.4876 (18)	C14—H14	0.9500
C2—C3	1.385 (2)	C15—C16	1.363 (2)
C2—C7	1.3850 (18)	C15—H15	0.9500
C3—C4	1.3836 (19)	C16—C17	1.4191 (19)
C3—H3	0.9500	C16—H16	0.9500
C4—C5	1.3825 (19)	C17—C18	1.419 (2)
C4—H4	0.9500	C18—C19	1.363 (2)
C5—C6	1.382 (2)	C18—H18	0.9500
C6—C7	1.3844 (19)	C19—C20	1.409 (2)
C6—H6	0.9500	C19—H19	0.9500
C7—H7	0.9500	C20—H20	0.9500
C11—C20	1.373 (2)		
			
O4—N1—O3	123.10 (12)	C13—C12—C17	118.61 (12)
O4—N1—C5	118.41 (13)	C13—C12—C11	123.94 (12)
O3—N1—C5	118.49 (12)	C17—C12—C11	117.43 (12)
C1—O1—C2	118.78 (10)	C14—C13—C12	120.47 (14)
O2—C1—O1	123.12 (12)	C14—C13—H13	119.8
O2—C1—C11	126.58 (11)	C12—C13—H13	119.8
O1—C1—C11	110.27 (11)	C13—C14—C15	120.82 (15)
C3—C2—C7	122.17 (12)	C13—C14—H14	119.6
C3—C2—O1	121.92 (11)	C15—C14—H14	119.6
C7—C2—O1	115.80 (12)	C16—C15—C14	120.24 (14)
C4—C3—C2	118.86 (12)	C16—C15—H15	119.9
C4—C3—H3	120.6	C14—C15—H15	119.9
C2—C3—H3	120.6	C15—C16—C17	120.72 (14)
C5—C4—C3	118.75 (13)	C15—C16—H16	119.6
C5—C4—H4	120.6	C17—C16—H16	119.6
C3—C4—H4	120.6	C18—C17—C16	120.73 (13)
C4—C5—C6	122.62 (12)	C18—C17—C12	120.13 (12)
C4—C5—N1	118.84 (13)	C16—C17—C12	119.13 (13)
C6—C5—N1	118.53 (12)	C19—C18—C17	120.82 (13)
C5—C6—C7	118.57 (12)	C19—C18—H18	119.6
C5—C6—H6	120.7	C17—C18—H18	119.6
C7—C6—H6	120.7	C18—C19—C20	119.87 (13)
C6—C7—C2	118.99 (13)	C18—C19—H19	120.1
C6—C7—H7	120.5	C20—C19—H19	120.1
C2—C7—H7	120.5	C11—C20—C19	121.17 (12)
C20—C11—C12	120.56 (12)	C11—C20—H20	119.4
C20—C11—C1	118.54 (11)	C19—C20—H20	119.4
C12—C11—C1	120.86 (12)		
			
C2—O1—C1—O2	2.09 (18)	C20—C11—C12—C13	−178.63 (13)
C2—O1—C1—C11	−176.10 (10)	C1—C11—C12—C13	3.73 (19)
C1—O1—C2—C3	−51.49 (16)	C20—C11—C12—C17	−0.49 (18)
C1—O1—C2—C7	132.16 (12)	C1—C11—C12—C17	−178.13 (11)
C7—C2—C3—C4	−2.4 (2)	C17—C12—C13—C14	0.4 (2)
O1—C2—C3—C4	−178.48 (12)	C11—C12—C13—C14	178.56 (14)
C2—C3—C4—C5	1.1 (2)	C12—C13—C14—C15	−0.8 (2)
C3—C4—C5—C6	0.5 (2)	C13—C14—C15—C16	0.4 (3)
C3—C4—C5—N1	179.64 (12)	C14—C15—C16—C17	0.3 (2)
O4—N1—C5—C4	−174.87 (13)	C15—C16—C17—C18	179.92 (14)
O3—N1—C5—C4	4.5 (2)	C15—C16—C17—C12	−0.7 (2)
O4—N1—C5—C6	4.29 (19)	C13—C12—C17—C18	179.70 (12)
O3—N1—C5—C6	−176.38 (13)	C11—C12—C17—C18	1.46 (18)
C4—C5—C6—C7	−0.9 (2)	C13—C12—C17—C16	0.31 (19)
N1—C5—C6—C7	179.98 (12)	C11—C12—C17—C16	−177.93 (12)
C5—C6—C7—C2	−0.3 (2)	C16—C17—C18—C19	178.26 (14)
C3—C2—C7—C6	2.0 (2)	C12—C17—C18—C19	−1.1 (2)
O1—C2—C7—C6	178.32 (12)	C17—C18—C19—C20	−0.2 (2)
O2—C1—C11—C20	−138.43 (14)	C12—C11—C20—C19	−0.8 (2)
O1—C1—C11—C20	39.68 (15)	C1—C11—C20—C19	176.85 (12)
O2—C1—C11—C12	39.26 (19)	C18—C19—C20—C11	1.2 (2)
O1—C1—C11—C12	−142.63 (11)		

### Hydrogen-bond geometry (Å, °)

**Table:**

*D*—H···*A*	*D*—H	H···*A*	*D*···*A*	*D*—H···*A*
C18—H18···O2^i^	0.95	2.45	3.3728 (18)	164

Symmetry codes: (i) *x*, −*y*+3/2, *z*−1/2.
